# Draft Genome Sequence of *Alcaligenes faecalis* BDB4, a Polyaromatic Hydrocarbon-Degrading Bacterium Isolated from Crude Oil-Contaminated Soil

**DOI:** 10.1128/genomeA.01346-17

**Published:** 2017-11-30

**Authors:** L. Paikhomba Singha, Rhitu Kotoky, Piyush Pandey

**Affiliations:** Department of Microbiology, Assam University, Silchar, Assam, India

## Abstract

*Alcaligenes fecalis* BDB4 was isolated from crude oil-contaminated soil in India. The genome sequence of *A. faecalis* BDB4 revealed the presence of important genes required for polyaromatic hydrocarbon (PAH) metabolism and other associated functions, such as chemotaxis, membrane transport, and biofilm formation, giving insight into the complete PAH mineralization potential of this bacterium.

## GENOME ANNOUNCEMENT

*Alcaligenes* spp. have been reported for their ability to degrade phenanthrene and various high-molecular-weight compounds and explored as potential degraders obtained from waste soil contaminated with solid waste oil and creosote samples (1–4). The complete mineralization potential of *Alcaligenes faecalis* toward polyaromatic hydrocarbons (PAHs) make it a promising candidate for bioremediation (5).

The bacterium *Alcaligenes faecalis* BDB4 was isolated from a crude oil-contaminated soil sample from West Bengal, India. It was found to utilize polyaromatic hydrocarbons (pyrene, chrysene and benzo[*a*]pyrene) as a sole source of carbon and energy. To determine the complete genome sequence of *Alcaligenes faecalis* BDB4, whole-genome shotgun sequencing was done using one Illumina paired-end library with an average insert size of ~400 bp. The paired-end sequencing libraries were prepared using an Illumina TruSeq Nano DNA library prep kit. The DNA was fragmented by using a Covaris M220, which generates double-stranded DNA (dsDNA) fragments with 3′ or 5′ overhang. The fragments were then subjected to end repair followed by adapter ligation to the fragments. The products were then PCR amplified with the index primer as described in the kit protocol and sequenced using NextSeq 500.

The sequenced raw data were processed to obtain high-quality clean reads using Trimmomatic v0.35 to remove ambiguous reads, adapter sequences, and low-quality sequences. These reads were trimmed using a quality score threshold of 20 and a length cutoff of 20 bp. Reference-guided assembly of the sample was performed using SAMtools. The procedure for genome annotation was done by using the Rapid Annotations using Subsystems Technology (RAST) server and the NCBI Prokaryotic Genome Annotation Pipeline (https://www.ncbi.nlm.nih.gov/genome/annotation_prok/) (6). The rRNA and tRNA genes were predicted and annotated using RNAmmer (7) and tRNAscan-SE (8), respectively. The genome of *Alcaligenes faecalis* BDB4 consists of one circular chromosome of 4,232,403 bp (50.25% GC content) and a plasmid of 4,576 bp.

The first version of the annotation includes 57 tRNA genes; 9 rRNA genes; and a total of 357 pseudogenes and 3,155 protein-coding genes involving pathways for xenobiotic biodegradation and metabolism, including catechol 1,2-dioxygenase, cytochrome P450 monooxygenase, etc., for toluene, xylene, dioxin, bisphenol, styrene, and naphthalene PAHs, and a complete operon for degradation of benzoate. Moreover, genes for membrane transport system, biofilm formation, quorum sensing, chemotaxis, and glutathione metabolism were also found.

### Accession number(s).

The complete genome sequences of *Alcaligenes faecalis* BDB4 and the plasmid pZD02 have been submitted to GenBank under the accession no. CP021883 and CP021884, respectively.
